# Secondary analysis of a randomised controlled trial on reducing sedentary behaviour and its effects on quality of life and wellbeing

**DOI:** 10.1038/s41598-025-20836-7

**Published:** 2025-10-22

**Authors:** Jooa Norha, Tanja Sjöros, Taru Garthwaite, Saara Laine, Kirsi Laitinen, Noora Houttu, Henri Vähä-Ypyä, Harri Sievänen, Eliisa Löyttyniemi, Tommi Vasankari, Juhani Knuuti, Kari K. Kalliokoski, Ilkka H. A. Heinonen

**Affiliations:** 1 Turku PET Centre, University of Turku and Turku University Hospital, P.O. Box 52, Turku, 20521 Finland; 2 Integrative Physiology and Pharmacology Unit, Institute of Biomedicine, University of Turku, Turku, Finland; 3 Nutrition and Food Research Center, University of Turku, Turku, Finland; 4 The UKK Institute, Tampere, Finland; 5 Department of Biostatistics, University of Turku, Turku University Hospital, Turku, Finland; 6 Faculty of Medicine and Health Technology, University of Tampere, Tampere, Finland

**Keywords:** Sedentary behaviour, Wellbeing, Quality of life, Accelerometer, Obesity, Human behaviour, Risk factors, Clinical trial design

## Abstract

High sedentary behaviour (SB) associates with poorer wellbeing among adults at increased cardiovascular risk. The aim of this study was to investigate the effects of reducing SB on perceived quality of life, depressive symptoms, stress, and workability among physically inactive adults with metabolic syndrome. Sixty-four sedentary, inactive adults [58 (SD 7) years old, 58% women, BMI 32 (4) kg/m^2^] with metabolic syndrome were randomised into the intervention (*n* = 33) and control (*n* = 31) groups. The 6-month intervention aimed at reducing SB by 1 h/day without adding exercise, while the control group kept their usual SB. All participants wore accelerometers throughout the study to monitor SB and physical activity. Health-related quality of life, stress, depressive symptoms and workability were assessed at baseline, at three months, and after the intervention using the RAND-36, perceived stress questionnaire (PSQ), general health questionnaire (GHQ-12), and the workability score. Main intervention effects were analysed using linear mixed models for repeated measurements. The RAND-36 vitality score decreased in the control group [-2.9 (95% CI -7.7, 1.9) and -1.0 (-5.9, 4.0) from baseline to three and six months, respectively] and increased in the intervention group [+5.7 (1.1, 10.4) and +5.3 (0.4, 10.3) from baseline to three and six months, respectively; between-group difference at three months *p* = 0.012 and at six months *p* = 0.079]. The PSQ index increased among all participants [+0.03 (-0.01, 0.06) from baseline to six months; time *p* = 0.033, no significant between-group differences *p* = 0.143]. Among all participants, the change in SB correlated negatively with the change in RAND-36 social functioning score, and the change in light physical activity correlated negatively with the change in PSQ index. Moreover, the change in SB breaks correlated negatively with the RAND-36 physical and social functioning scores. Aiming to reduce SB by 1 h/day may increase perceived vitality among physically inactive adults with metabolic syndrome.

The study was pre-registered at Clinicaltrials.gov (5/4/2017, NCT03101228).

**Supplementary Information:**

The online version contains supplementary material available at 10.1038/s41598-025-20836-7.

## Introduction

Cardiovascular risk factors, such as metabolic syndrome, type II diabetes, obesity, and physical inactivity are highly prevalent, affecting around 30% of Western adults^1,2^. As expected, most incident cardiovascular disease can be attributed to common risk factors^3^ – however, less emphasis is placed on the perceived quality of life and wellbeing-related consequences of an adverse cardiovascular risk profile. Wellbeing can be defined as experiencing positive feelings and meeting full potential as a member of a society^4^, and adverse perceptions, such as depressive symptoms, stress, and reduced workability, can therefore decrease wellbeing. Furthermore, quality of life is partially an overlapping concept with wellbeing, but wellbeing includes also a more subjective perception^4^. Importantly, the presence of cardiovascular risk factors associates with poorer quality of life^5,6^. Moreover, better perceived wellbeing, including positive emotions, optimism, and life satisfaction, seems to lead to better cardiovascular outcomes via promoting optimal physiological functioning (e.g., improved immune function, reduced cortisol, and decreased blood pressure), improving health behaviour, and protecting from the adverse health effects of stress^7^. Thus, ways to improve quality of life and wellbeing would be essential, especially for individuals with existing risk factors.

Multiple observational and interventional studies show that a higher level of physical activity (PA) associates with and leads to better self-rated wellbeing and quality of life in adults^8–10^. Moreover, a recent network meta-analysis of 218 studies concluded that physical exercise, especially walking or jogging, is an effective treatment for clinical depression^11^. Conversely, high sedentary behaviour (SB; defined as any awake sitting, reclining or lying activities with < 1.5 metabolic equivalent [MET] energy consumption) associates with poorer wellbeing and quality of life across different adult populations from healthy < 40 year-old men to middle-aged women and individuals with prediabetes or type II diabetes^9,12–14^. SB may be especially detrimental for wellbeing if it replaces moderate-to-vigorous PA (MVPA)^15^. As SB also associates with increased cardiovascular risk factors^16^, which further associate with poorer wellbeing and quality of life^5,6^, reducing SB would be an interesting target to improve wellbeing and the quality of life among individuals with high risk.

However, studies on the effects of reducing SB on self-rated wellbeing and quality of life are scarce, especially among physically inactive adults with metabolic syndrome. However, a cluster randomized controlled trial aiming to reduce occupational SB reported beneficial effects on job performance, recovery from occupational fatigue, anxiety, and psychological and overall quality of life among 146 healthy desk workers aged 41 years old^17^.

We have conducted a randomised controlled trial among physically inactive adults with metabolic syndrome, where the intervention group aimed at reducing daily SB by 1 h for six months. Based on previous intervention studies, the 1 h target has been observed to be an achievable reduction in SB^18,19^. The primary outcome of our study was whole-body insulin sensitivity^20^. In terms of cardiometabolic health, the intervention had minor effects at best, as reviewed previously^21^. However, we did observe beneficial effects on back pain^22^, which raises the question of whether other subjective outcomes could be influenced by the SB reducing intervention. Thus, in this secondary analysis of the randomised controlled trial, the aim was to investigate the effects of reducing SB on perceived quality of life, depressive symptoms, stress, and workability among physically inactive adults with metabolic syndrome. Additionally, as explorative analyses, we investigated the cross-sectional associations between cardiometabolic health and the quality of life and wellbeing outcomes, and whether the changes in SB, PA, or body composition during the intervention associate with the perceived quality of life, depressive symptoms, stress, and workability.

## Materials and methods

The data used in this study consists of secondary outcomes of a six-month randomized controlled trial the main aim of which was to investigate the effects of reducing SB on whole-body insulin sensitivity^20^. The study was pre-registered at Clinicaltrials.gov (5/4/2017, NCT03101228), and no major amendments to the protocol were performed after registration. It was conducted at the Turku PET Centre (Turku, Finland) between April 2017 and March 2020 according to the Declaration of Helsinki. The Ethics Committee of the Hospital District of Southwest Finland approved the study (16/1801/2017). All participants gave informed consent before entering the study. As this is an analysis of the secondary outcomes of the trial, most of the study methods have been reported in more detail earlier^20,23–25^.

### Participants and recruitment

Participants were recruited from the local community using newspaper and bulletin leaflet advertisements between April 2017 and August 2019. The inclusion criteria were self-reported physical inactivity (< 120 min of moderate PA per week as defined in an initial interview), high SB (≥ 10 h/day or ≥ 60% of accelerometer wear time during screening), metabolic syndrome^26^, age 40–65 years, and body mass index (BMI) 25–40 kg/m^2^. The exclusion criteria were diagnosed diabetes or fasting blood glucose ≥ 7 mmol/l, history of cardiac disease, diagnosed depressive or bipolar disorder, or any other condition that could hinder the study protocol.

The study consisted of two phases: a four-week screening phase to assess eligibility and baseline activity behaviours and a subsequent six-month intervention period. The outcome measures were assessed before the intervention period, at the midpoint of the intervention (i.e., three months), and finally after the intervention (at six months).

### Intervention

Eligible participants (*n* = 64) were randomized into the intervention (INT, *n* = 33) and control (CON, *n* = 31) groups using 1:1 random permuted block randomization (block size 44) stratified by sex. The randomization was performed by a statistician using SAS 9.4 (SAS Institute Inc., Cary, NC, USA).

The aim of the intervention group was to reduce daily SB by 1 h for six months. The target of 1 h was chosen as it has been observed to be an achievable reduction in SB^18,19^. The ways to reduce SB were discussed individually with a physiotherapist during a one-hour counselling session, and non-exercise activities, such as using standing desks, walking while talking on the phone, or interrupting SB by lightly walking, were encouraged. The control participants were advised to maintain their usual activity behaviour. To facilitate adherence to the intervention or control, all participants wore accelerometers throughout the study, and the device was connected to a mobile application (ExSed, UKK Terveyspalvelut Oy, Tampere, Finland) which the participants could use to monitor their daily activities in relation to the individually set goals. In the intervention group, the goals for SB and PA were set so that 1 h was subtracted from the mean daily SB during screening and subsequently, 1 h was added to light PA (LPA), MVPA, or standing based on individual preference. However, a maximum of 20 min was added to MVPA. The goals were set during the individual counselling session with the physiotherapist. In the control group, the goals were set equal to the measured behaviours during screening.

### Accelerometry

All participants were given triaxial accelerometers to be worn on the right hip for four weeks during screening (UKK AM30, UKK Terveyspalvelut Oy, Tampere, Finland) and for the whole six-month intervention period (Movesense, Suunto, Vantaa, Finland). During the screening, the device was worn on an elastic belt, and during the intervention the device was clipped onto the waistband of the clothes. The participants were advised to wear the device during waking hours except when the device could be exposed to water.

The accelerometer data was collected with 52 Hz frequency, ± 8 gravity unit range, and 4 milligravity resolution. The raw data was analysed in six-second epochs using validated mean amplitude deviation (MAD, for activity intensity) and angle for posture estimation (APE, to differentiate SB and standing) methods^27,28^. Activities corresponding to 1.5 to < 3.0 MET were classified as LPA and ≥ 3.0 MET as MVPA. When the MAD was < 1.5 MET, the APE algorithm was used to classify the posture into standing or SB based on the device’s inclination angle in reference to the Earth’s gravity vector during walking. Additionally, the step count and the number of breaks in SB were calculated using the previously published algorithms^27^. In brief, steps were detected from the vertical component of the acceleration during detected PA, and breaks in SB were recorded if the 1-min moving average of the estimated MET value was < 1.5 and a clear vertical acceleration with subsequent movement or standing was detected^27^.

A minimum of four days with > 10 h/day measurement was considered valid. This threshold was chosen in order to capture most of the PA behaviour during the measurement without sacrificing the number of valid days too much^29^. Measured time exceeding 19 h/day was subtracted from the measured SB as it likely meant the participant had worn the device while sleeping.

### Questionnaires for health-related quality of life, depressive symptoms, stress, and workability

Health-related quality of life was assessed using the validated Finnish version of the RAND 36-Item Health Survey 1.0 (RAND-36)^30^. The RAND-36 provides eight subscales of different quality of life related factors: physical functioning, physical role functioning, emotional role functioning, vitality (or energy/fatigue), emotional well-being, social functioning, bodily pain, and general health. The questionnaire consists of 36 questions which the participant scores on 2–6 point Likert-scales. The scores of the individual items are first recoded and then averaged according to the instructions to provide a score of 0–100 for the eight subcategories, with a higher value representing a more favourable health state.

Depressive symptoms over the last weeks were assessed using the validated Finnish version of the General Health Questionnaire-12 (GHQ-12)^31^. The 12 items of the questionnaire with a four-point Likert-scale were scored using the standard method such that an answer in either of the last two categories (“worse or much worse than usual”) was scored as one point. The GHQ score was then calculated as the total sum, yielding a score of 0–12, where a higher value represents more depressive symptoms^32^. The validity of the GHQ-12 is acceptable for detecting depressive disorder in the general population^32^.

Perceived stress was assessed using a Finnish translation of the Perceived Stress Questionnaire (PSQ), which is a validated tool for measuring stress over the last month^33^. The PSQ consists of 30 questions with four-point Likert-scales. To calculate the PSQ index, eight of the item scores are first inverted. Then, the PSQ index is calculated as (raw score–30)/90, yielding an index between 0 and 1, where a higher score indicates higher stress^33^.

Workability was assessed using the Finnish version of the single-item workability score, which is a simple and valid tool to predict sick leaves (a marker of workability) and health-related quality of life, and it correlates strongly with the more extensive multi-item workability index^34,35^. The participants were asked to rate their current workability on a scale from 0 to 10, where 10 indicates the best workability and 0 indicates incapacity for work.

### Cardiometabolic measures

Height was measured using a wall-mounted stadiometer and weight was measured using the Bod Pod device (Bod Pod, COSMED USA Inc., Chicago, IL, USA). BMI was calculated as body weight (kg) divided by height (m) squared. Body fat percentage was measured using validated air displacement plethysmography (Bod Pod, COSMED USA Inc., Chicago, IL, USA) after at least a four-hour fast. Blood biochemistry analyses for cardiometabolic risk factors and maximal exercise testing are described in the Supplementary file.

### Statistical analysis

Baseline characteristics are presented as mean (standard deviation), n (%), or median (interquartile range), as appropriate.

The main analyses for intervention effects (i.e., time and group comparisons) were performed using linear mixed models for repeated measurements, and model-based means with 95% confidence intervals are reported. Intervention effects on the outcomes (quality of life, depressive symptoms, stress, and workability) were analysed as changes from baseline to 3 months and baseline to 6 months. This approach was chosen to ensure the normal distribution of the residuals in the linear mixed models, as the residuals were not normally distributed when analysed using the absolute values, despite variable transformations. The distribution of the residuals was evaluated visually. The main analyses included group, time and group x time as the independent variables and the models were adjusted for the baseline value of the outcome variable and sex. Compound symmetry covariance structure for time was used and pairwise comparisons were adjusted using the Tukey-Kramer method. Group differences were estimated for each time point. This analysis method can include all data regardless of missing values. The analyses were performed in SAS 9.4. (SAS Institute Inc., Cary, NC, USA).

As an additional analysis, we analysed the correlations between the changes in activity behaviour, body composition, and the changes in the quality of life, depressive symptoms, stress, and workability measures using Spearman’s correlation among all study participants (regardless of group allocation). Both three and six-month changes in the questionnaire outcomes were included in the correlation analyses. This was done regardless of the original groups to assess the relationship between activity behaviour and the quality of life and wellbeing-related data. Moreover, the cross-sectional correlations at baseline between the quality of life and wellbeing-related measures, activity behaviour, and cardiometabolic risk factors were analysed using Spearman’s correlation as an explorative analysis. The correlation analyses were performed in IBM SPSS Statistics 28.0 (IBM Corp., Armonk, NY, USA).

P-value of < 0.05 (two-tailed) was considered statistically significant. The sample size for this study was calculated for the primary outcome of the whole trial, which was whole-body insulin sensitivity^20^, and thus no power calculations for the questionnaire data used in this study were performed.

## Results

**Fig. 1:**
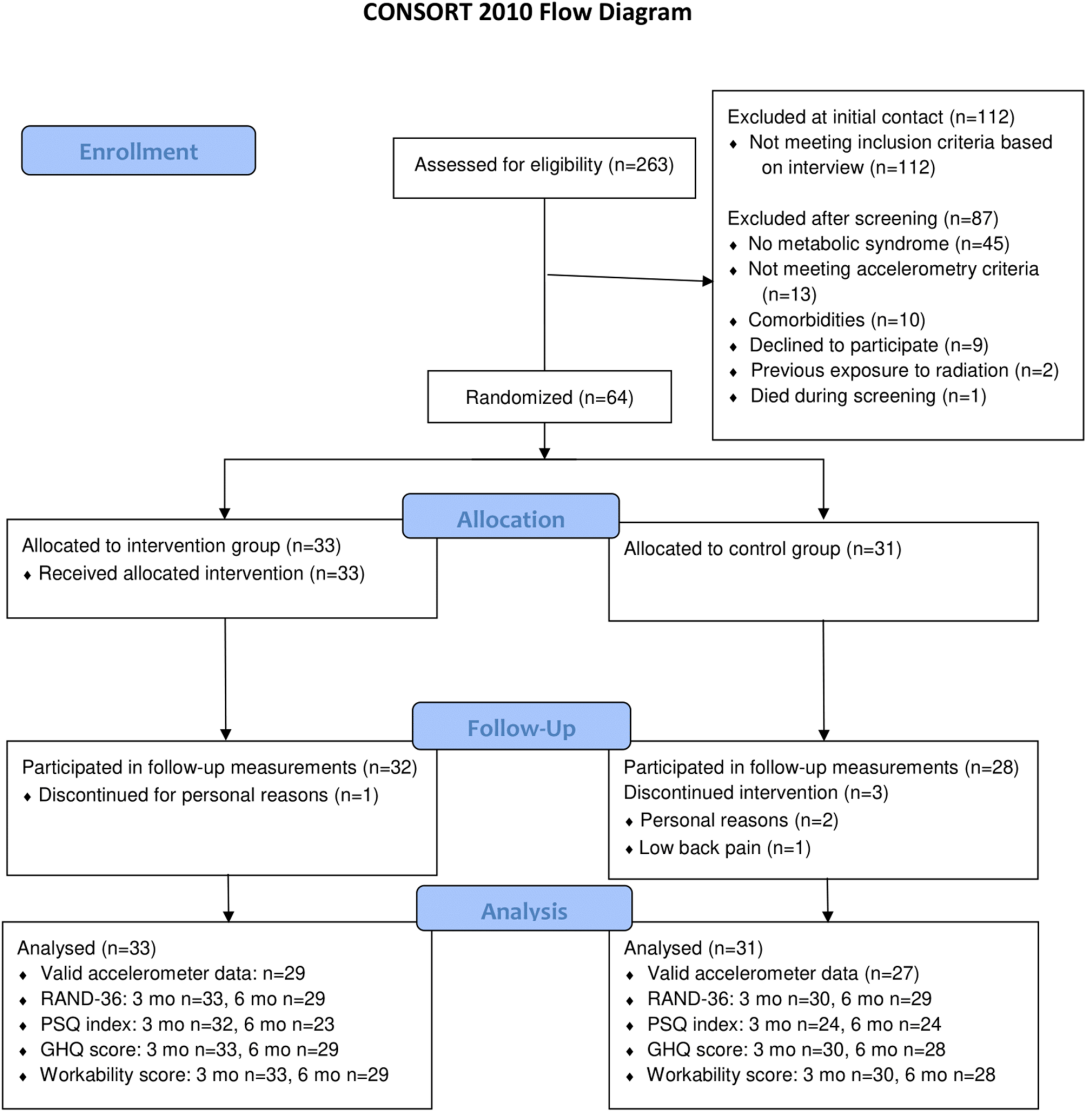
CONSORT Flow Diagram.

Initially, 263 volunteers were contacted, 151 of whom were screened to fulfil the target of 64 eligible participants who were then randomized into the INT (*n* = 33) and CON (*n* = 31) groups. One participant in the INT group and three participants in the CON group discontinued due to personal reasons or low back pain unrelated to the study (see Fig. 1 for the CONSORT Flow Diagram). The baseline characteristics are presented in Table 1. The baseline correlations between the questionnaire data and activity behaviour, body composition, and cardiometabolic risk factors are presented in Supplementary Table 1. As expected, some correlations indicating mostly poorer quality of life and wellbeing with adverse cardiometabolic health were observed.

**Table 1:** Baseline characteristics of the intervention and control groups. Presented as mean (standard deviation) if not otherwise stated.

	Intervention (*n* = 33)	Control (*n* = 31)
Sex, n of females (%)	20 (61)	17 (55)
Age, years	59 (6)	57 (8)
BMI, kg/m^2^	31.5 (4.0)	31.7 (4.6)
Body fat percentage	43.1 (8.0)	43.1 (8.0)
**RAND-36**		
Physical functioning*	90 (20)	90 (16)
Role functioning: physical*	100 (13)	100 (25)
Role functioning: emotional*	100 (0)	100 (33)
Vitality*	75 (23)	75 (35)
Emotional well-being*	84 (14)	80 (12)
Social functioning*	100 (13)	88 (25)
Bodily pain*	90 (33)	78 (33)
General health*	70 (20)	69 (20)
PSQ index*	0.19 (0.11)	0.26 (0.27)
Workability score	8 (1)	8 (1)
GHQ score*	0 (1)	0 (2)
**Accelerometry**		
Measurement duration, days	25.8 (3.7)	25.7 (3.4)
Accelerometer wear time, h/day	14.5 (1.0)	14.6 (1.0)
Sedentary time, h/day	10.0 (0.9)	10.1 (1.1)
Standing time, h/day	1.8 (0.6)	1.8 (0.6)
LPA, h/day	1.7 (0.4)	1.8 (0.5)
MVPA, h/day	1.0 (0.3)	1.0 (0.3)
Steps/day	5203 (1910)	5091 (1760)
Breaks in SB/day	28 (8)	29 (8)

*Median (interquartile range).

### Intervention

### Accelerometry

As reported earlier in more detail^20^, the INT group reduced SB by 40 min/day, on average, and increased MVPA by 20 min/day. In the CON group SB and MVPA remained unchanged. Step count increased in both groups, but the increase was statistically significantly higher in the INT group (an increase of 3300 vs. 1600 steps/day in the INT and CON groups, respectively). LPA increased by 10 min/day, on average, without statistically significant between-group differences. No statistically significant changes in standing were observed.

### Quality of life, depressive symptoms, stress, and workability

**Fig. 2:**
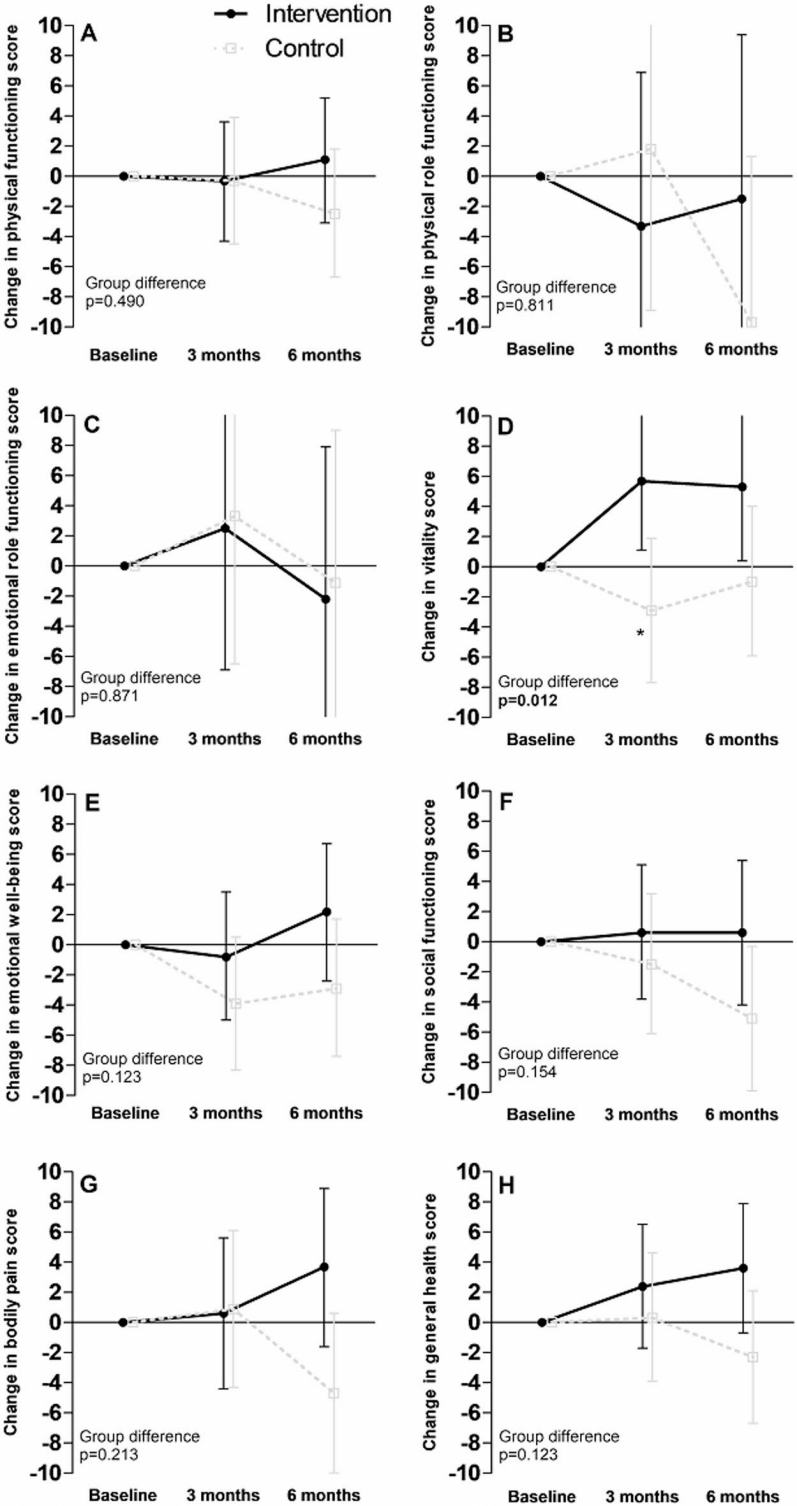
Changes in the RAND-36 subscales in the intervention and control groups at three and six months. Panel **A** physical functioning; **B** physical role functioning; **C** emotional role functioning; **D** vitality; **E** emotional well-being; **F** social functioning; **G** bodily pain; **H** general health. Presented as model-based means and 95% confidence intervals. *Between-group difference at three months *p* = 0.012.

The changes in RAND-36 scores are presented in Fig. 2. During the whole intervention, the changes in vitality score were statistically significantly different between groups (*p* = 0.012), with the mean score increasing in the INT [+5.7 (95% CI 1.1, 10.4) and +5.3 (0.4, 10.3) from baseline to three and six months, respectively] and decreasing in the CON group [-2.9 (-7.7, 1.9) and -1.0 (-5.9, 4.0) from baseline to three and six months, respectively]. However, the between-group difference was significant only at three months (*p* = 0.012) and not at six months (*p* = 0.079). No statistically significant changes in any other subcategories of the RAND-36 were observed.

**Fig. 3:**
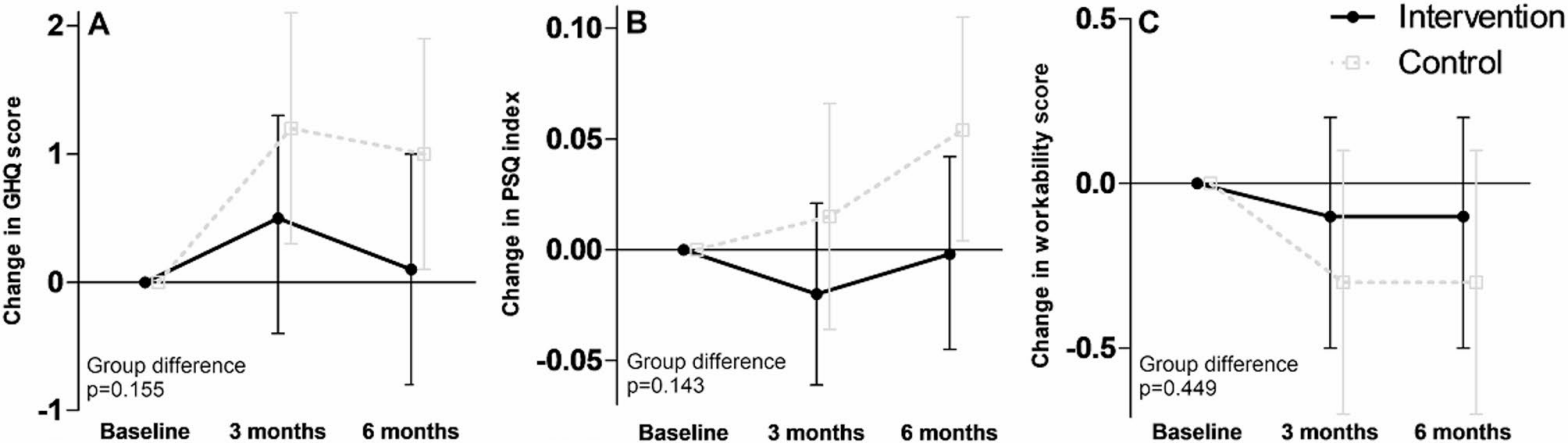
Changes in the general health questionnaire (GHQ) score (**A**), perceived stress questionnaire (PSQ) index (**B**), and workability score (**C**) in the intervention and control groups at three and six months. Presented as model-based means and 95% confidence intervals. Panel B, perceived stress index: time *p* = 0.033.

The changes in the PSQ index, GHQ score and workability score are presented in Fig. 3. No statistically significant differences between the groups were observed in these outcomes. However, the PSQ index increased in the whole study sample [+0.03 (-0.01, 0.06) from baseline to six months; time *p* = 0.033), and this was mainly driven by the increases in the CON group, although no statistically significant between-group differences were observed (group *p* = 0.143).

The numerical estimates and all p-values corresponding to Figs. 2 and 3 are presented in Supplementary Table 2.

### Correlations of changes

The correlations between the changes in activity behaviour, body composition, and the changes in the questionnaire variables are presented in Table 2. The change in SB correlated inversely with the change in social functioning (ρ = -0.23, *p* = 0.02). Additionally, the change in LPA correlated inversely with the change in PSQ index (ρ = -0.24, *p* = 0.039). Although not statistically significant, a positive trend between the change in MVPA and the change in general health was observed (ρ = 0.19, *p* = 0.059). Finally, the change in SB breaks correlated inversely with the change in physical and social functioning (ρ = -0.22 and -0.23, respectively, *p* < 0.03 for both).

**Table 2:** Correlations (ρ) between the changes (Δ) in activity behaviour, body composition and the changes in the questionnaire data.

		Δ SB	Δ Standing	Δ LPA	Δ MVPA	Δ Steps	Δ Breaks	Δ BMI	Δ Body fat %
Δ Physical functioning	ρ	0.10	0.03	-0.08	-0.05	-0.05	**-0.22** ^*^	**-0.19** ^*^	-0.11
	P-value	0.326	0.767	0.442	0.621	0.639	**0.027**	**0.037**	0.221
Δ Role functioning: physical	ρ	0.08	-0.01	-0.14	-0.08	-0.10	-0.07	-0.05	-0.05
	P-value	0.438	0.885	0.161	0.448	0.305	0.464	0.608	0.587
Δ Role functioning: emotional	ρ	-0.07	0.12	0.12	0.01	-0.12	-0.16	0.14	-0.05
	P-value	0.519	0.243	0.232	0.933	0.217	0.123	0.129	0.607
Δ Vitality	ρ	-0.15	0.04	0.16	0.05	-0.05	-0.18	**-0.25** ^*^	-0.10
	P-value	0.147	0.683	0.101	0.615	0.609	0.071	**0.007**	0.286
Δ Emotional well-being	ρ	-0.12	0.08	0.08	0.00	0.07	-0.02	-0.08	-0.13
	P-value	0.218	0.407	0.447	0.996	0.468	0.834	0.412	0.150
Δ Social functioning	ρ	**-0.23** ^*^	0.18	0.19	0.10	-0.14	**-0.23** ^*^	0.02	-0.14
	P-value	**0.020**	0.079	0.053	0.337	0.171	**0.023**	0.794	0.116
Δ Pain	ρ	-0.04	0.03	0.06	0.12	0.08	-0.12	**-0.21** ^*^	**-0.24** ^*^
	P-value	0.659	0.751	0.569	0.231	0.420	0.243	**0.023**	**0.008**
Δ General health	ρ	-0.17	0.02	0.16	0.19	0.04	-0.07	0.04	**-0.19** ^*^
	P-value	0.091	0.808	0.106	0.059	0.684	0.474	0.682	**0.038**
Δ PSQ index	ρ	0.19	0.07	**-0.24** ^*^	-0.12	-0.09	0.02	0.06	0.06
	P-value	0.108	0.544	**0.039**	0.314	0.471	0.836	0.607	0.601
Δ Workability score	ρ	-0.14	-0.04	0.18	0.13	0.15	0.04	**-0.28** ^*^	-0.12
	P-value	0.178	0.689	0.081	0.201	0.146	0.703	**0.002**	0.181
Δ GHQ score	ρ	0.09	0.04	-0.04	-0.10	0.02	-0.01	-0.04	0.05
	P-value	0.375	0.703	0.701	0.337	0.880	0.931	0.639	0.593

SB = sedentary behaviour, LPA = light physical activity, MVPA = moderate-to-vigorous physical activity, BMI = body mass index, PSQ = Perceived stress questionnaire (higher score indicates higher stress), GHQ = General health questionnaire (higher score indicates more depressive symptoms). *Marks statistically significant (*p* < 0.05) correlations.

The changes in BMI correlated inversely with the changes in physical functioning, vitality, bodily pain, and the workability score (ρ = -0.19, -0.25, -0.21, -0.28, respectively, *p* < 0.05 for all). Moreover, the changes in body fat percentage correlated inversely with the changes in bodily pain and general health perceptions (ρ = -0.24 and − 0.19, respectively, *p* < 0.05 for both).

## Discussion

In this study, although we show that reducing SB may have positive effects on perceived vitality (or energy) measured using the RAND-36 health-related quality of life questionnaire, the intervention generally did not have significant effects on other aspects of health-related quality of life, depressive symptoms, stress, or workability. Nevertheless, in the secondary analyses, we show that changes in activity behaviours associate with changes in social and physical functioning, and stress.

Previously, a secondary analysis of a six-month randomized controlled trial among 24 adults with back pain reported improved vitality with reduced SB^36^, similar to the current study. In the context of the RAND-36, vitality refers to feeling energetic and full of pep, and not feeling worn-out or tired. Moreover, beneficial effects on social functioning and bodily pain scores were found in that study^36^, whereas we did not observe any significant changes in these. However, albeit measured with questionnaires rather than accelerometers, the previous intervention induced a 1.5 h/day reduction in SB^36^, which is two times more than in our study. Furthermore, a randomised crossover trial of 28 office workers found that replacing sitting at work with standing (using standing desks) increased feelings of being energetic assessed by ecological momentary assessment questions that have to be answered in the moment twice daily^37^.

Findings from laboratory-based short-term studies provide possible physiological explanations for the improved vitality. Interrupting prolonged sitting improves working memory, increases serum brain-derived neurotrophic growth factor, and cerebral blood flow^38,39^. Additionally, cross-sectional findings suggest that high SB associates with greater fatigue and sleep problems^40,41^. Combined, these findings support our result that SB plays a role in perceived vitality or energy throughout the day. Moreover, we have previously reported that feeling tired after work associates with less leisure-time PA^42^. Therefore, as reducing SB has the potential to increase vitality – in other words, reduce tiredness – a SB reduction intervention may have the potential to increase leisure-time PA.

What is more, a previous study reported that an intervention reducing occupational SB reduced work-related fatigue and improved the quality of life^17^. However, even though our finding of improved vitality parallels the finding of reduced work-related fatigue, we did not observe any statistically significant differences in other aspects of health-related quality of life measured with the RAND-36. The previous study reporting improved environmental, psychological, and overall quality of life used the World Health Organization Quality of Life-BREF tool^17^. While the different measurement tools and, subsequently, different subscales may explain the difference in findings, the sample size may also play a role. In our study, emotional wellbeing (similar to the psychological quality of life) trended slightly in favour of the intervention group, however, this was not statistically significant (*p* = 0.123). Therefore, it is possible that our study (*n* = 64) was underpowered to detect a statistically significant difference, whereas the previous study had a larger sample size (*n* = 146) and, with that, more statistical power.

In addition to the tentative physiological mechanisms for improved vitality discussed briefly above, the behavioural nature of the intervention may also explain why a SB reduction intervention could improve vitality. Individually tailoring the intervention and using self-monitoring to meet the goals are effective ways to support empowerment, which in turn may lead to positive health outcomes^43,44^. However, it could be argued that also succeeding in the control group’s goals of maintaining baseline PA behaviour could induce such feelings of empowerment. As depressive symptoms associate with fatigue^45^, it could be speculated that the positive emotions and a sense of empowerment from achieving the behavioural change (i.e., SB reduction) could reduce fatigue or increase vitality. This could be especially true with the intervention where the participants were able to self-monitor the achievement of their daily SB and PA goals. The vitality score in the RAND-36 consists of four statements that the participants rate on a scale from 1 to 6, and the statements address feeling “full of pep”, having a lot of energy, feeling worn out and tiredness, the latter two which may be interpreted as aspects of fatigue.

However, in the explorative correlation analyses, we did not find a correlation between the change in SB and the change in vitality among all participants (ρ = -0.15, *p* = 0.147). While the estimate is towards the expected direction, this further supports that the behavioural nature of the intervention could also play a role in the improvement of vitality with the SB-reducing intervention.

In the explorative correlation analyses among all study participants, we observed that improved social functioning associated with less SB and less breaks in SB. Improved social functioning associated non-significantly with increased LPA (ρ = 0.19, *p* = 0.053). These are in line with the previous finding of a SB-reducing intervention improving social functioning among adults who have a desk-based occupation and chronic low back pain^36^. The social functioning in RAND-36 is assessed by two statements on the degree and frequency of bodily or emotional health affecting social functioning. Taken together, it seems possible that reducing SB and subsequently reducing the number of breaks in SB (due to less SB to interrupt) could improve social functioning or vice versa. Moreover, a reduction in SB breaks associated with improved physical functioning. Although speculative, we believe that decreasing the frequency of SB interruptions would not lead to better physical functioning. Instead, if the individual spends overall less time in SB, and as a result, SB is interrupted less frequently, physical functioning could improve. However, as the finding is only a correlation, it may also be that decreased physical functioning (e.g., due to pain) could lead to coping with a musculoskeletal complaint by more frequently changing body posture (i.e., breaking up SB)^25^. Finally, the change in the PSQ index correlated inversely with the change in LPA, meaning reduced stress when LPA is increased, or vice versa. It is well known that perceived stress associates with less PA ^46^, which highlights the importance of both managing stress and increasing PA to promote wellbeing. However, as the correlation coefficients in this study were mostly only below 0.3, they should be interpreted as preliminary evidence. Nevertheless, the correlations were mostly credible and consistent which would imply that the correlations are not false positives.

Given the known associations between cardiometabolic risk factors and perceived wellbeing^5–7^, it is not surprising that we observed increases in markers of body adiposity (BMI and body fat percentage) associating with decreased physical functioning, vitality, general health perceptions, workability and increased bodily pain. However, this highlights the benefits gained for perceived wellbeing when cardiometabolic health is improved.

The present findings should be interpreted with caution. While our findings were mainly reasonable and consistent, we did not observe statistically significant intervention effects on most of the quality of life and wellbeing measures. Nevertheless, in virtually all measures at three and six months, there was a tendency towards improvement in the intervention group and a decline in the control group. Taken together with the previous evidence on the benefits of PA on perceived quality of life and wellbeing^8–11^, this would suggest that the present study was underpowered to detect statistically significant differences in the variables of interest, especially as a non-exercise-based SB reduction intervention is likely lower intensity than a PA or exercise intervention. Therefore, more randomized controlled trials with adequate statistical power are needed. Moreover, unlike the current study, future studies should also include long-term follow-up measurements, as the intervention effects beyond the six months remain elusive in our study. Finally, the study sample should be acknowledged. The current findings cannot be generalized beyond physically inactive adults with metabolic syndrome.

Speculatively, in addition to the low statistical power, a possible explanation for the lack of intervention effects on most subcategories of the RAND-36, GHQ-12, PSQ, and workability score is the behaviour change in the control group. As reported previously^20^, the control group also increased their daily step count, albeit less than the intervention group. Moreover, the control group also slightly increased their MVPA during the last quartile of the intervention^20^. These increases in PA may have diluted any between-group differences in the outcomes of the current study. Indeed, it is known that simply providing a PA measurement device can increase PA and reduce SB, especially among late middle-aged adults with obesity^47^.

Another explanation for the number of null findings in this study relates to the methods for measuring quality of life, depressive symptoms, stress, and workability. Although the RAND-36, GHQ-12, PSQ, and workability score are meant to be used among the general population, it may be appreciated that the participants in this study had relatively good scores in all of them. For example, the GHQ score has a possible range of 0 to 12, and our participants had a median score of 0 in both groups, meaning no depressive symptoms. Similarly, the PSQ index was 0.19 and 0.26 in the intervention and control groups, respectively, when the index ranges from 0 to 1 and a lower score represents lower stress. Moreover, five out of eight RAND-36 subcategories scored 90 or higher in the intervention group, and the same was observed in three subcategories in the control group. Finally, the workability score was 8/10 in both groups. This means that the questionnaires used in this study were suboptimal for this sample. Floor- and ceiling effect (i.e., having close to minimum or maximum score which restricts changes within the scale) are therefore likely here.

Consequently, the only statistically significant intervention effect was observed in the vitality score, which had one of the least good scores at baseline (75 out of 100). However, the lowest score was observed in the general health perceptions subcategory of the RAND-36 (i.e., 70 and 69/100 in the intervention and control group, respectively). The reason we did not observe statistically significant changes in this subcategory could be related to the fact that the intervention had no effects on overall cardiometabolic health^21^, and that could be reflected in the general health perceptions, too.

The strengths of this study include the randomized controlled trial setting and the six-month duration with additional midpoint questionnaire evaluations. Moreover, the utilized questionnaires are commonly used and validated. Additionally, activity behaviour was measured using accelerometers throughout the study, and validated data processing algorithms were used. Indeed, the intervention was successful in reducing SB by 40 min/day, on average^20^.

## Conclusion

An intervention aimed at reducing daily SB by 1 h could increase perceived vitality among physically inactive adults with metabolic syndrome. Therefore, a SB reducing intervention could be useful for physically inactive adults with metabolic syndrome who wish to improve their vitality. In the future, the effects of reducing SB on perceived vitality could be investigated in specific populations with suboptimal vitality, such as individuals with sleep disturbances or fatigue. If our current results also apply in these populations, the SB reducing intervention could help in alleviating the lack of perceived vitality.

## Supplementary Information

Below is the link to the electronic supplementary material.

Supplementary Material 1

## Data Availability

Data collected during this study is available from the corresponding author upon a reasonable request.
