# Supplementary material for: Secondary analysis of a randomised controlled trial on reducing sedentary behaviour and its effects on quality of life and wellbeing
Source: Sci Rep. 2025 Oct 22;15:36861. doi: 10.1038/s41598-025-20836-7 (PMC12546735; doi:10.1038/s41598-025-20836-7)
Supplement: Supplementary file 1 — Supplementary Material 1 [file 41598_2025_20836_MOESM1_ESM.docx]

**Secondary analysis of a randomised controlled trial on reducing sedentary behaviour and its effects on quality of life and wellbeing**

**Supplementary material**

Jooa Norha^1^*, Tanja Sjöros^1^, Taru Garthwaite^1^, Saara Laine^1^, Kirsi Laitinen^2, 3^, Noora Houttu^2^, Henri Vähä-Ypyä^4^, Harri Sievänen^4^, Eliisa Löyttyniemi^5^, Tommi Vasankari^4, 6^, Juhani Knuuti^1^, Kari K. Kalliokoski^1^, Ilkka H. A. Heinonen^1^

1. Turku PET Centre, University of Turku and Turku University Hospital, Finland
2. Integrative Physiology and Pharmacology Unit, Institute of Biomedicine, University of Turku, Turku, Finland
3. Nutrition and Food Research Center, University of Turku, Turku, Finland
4. The UKK Institute, Tampere, Finland
5. Department of Biostatistics, University of Turku and Turku University Hospital, Finland
6. Faculty of Medicine and Health Technology, University of Tampere, Finland

*Corresponding author: Jooa Norha, Turku PET Centre, University of Turku and Turku University Hospital, P.O. Box 52, 20521 Turku, Finland, E-mail: [jooa.norha@utu.fi](mailto:jooa.norha@utu.fi)

**Table of contents**

Cardiovascular risk factor analysis methods.

Supplementary Table 1. Cross-sectional correlations at baseline among all study participants.

Supplementary Table 2. Model-based means (95% confidence intervals) of the changes in questionnaire scores at three and six months in the intervention and control groups.

**Cardiovascular risk factor analysis methods**

Maximal oxygen uptake was measured using a maximal bicycle ergometer test (eBike EL Ergometer with Case v6.7; GE Medical Systems Inc.) and direct respiratory gas measurements (Vyntus CPX, CareFusion), as described previously [1]. The protocol started at 25 W and the load was increased by 25 W every 3 minutes until criteria for maximal testing (respiratory exchange ratio >1.0, plateau in oxygen uptake despite increasing load, or heart rate ±10 beats/min of the age-predicted maximum) were achieved or unwillingness to continue the test by the participant. Maximal oxygen uptake was scaled to body mass and fat free mass.

Systolic and diastolic blood pressure were measured using a digital sphygmomanometer (Apteq AE701f, Rossmax International Ltd, Taipei, Taiwan) after at least a 10-min seated rest, and the average of 2-3 measurements was used, as described earlier [2].

Venous blood samples collected after at least 10 h of fasting were analysed at the Turku University Hospital Laboratory [3]. Plasma glucose was determined by enzymatic reference method with hexokinase GLUC3, and plasma triglycerides, total, low density and high density lipoprotein cholesterol by enzymatic colorimetric tests (Cobas 8000 c702, Roche Diagnostics GmbH, Mannheim, Germany). HbA1c was determined by turbidimetric inhibition immunoassay (Cobas 6000 c501, Roche Diagnostics GmbH, Mannheim, Germany).

Whole-body insulin sensitivity (M-value, μmol/min/kg) was assessed during hyperinsulinemic-euglycemic clamp by simultaneously infusing intravenous insulin (Actrapid, 100 U/ml, Novo Nordisk, Bagsvaerd, Denmark) and glucose, as described in more detail previously [4]. Blood samples were collected every 5 min during the clamp to titrate the glucose infusion rate to maintain a 5 mmol/l plasma glucose concentration.

**References**

[1] Norha J, Sjöros T, Garthwaite T, Laine S, Saarenhovi M, Kallio P, et al. Effects of reducing sedentary behavior on cardiorespiratory fitness in adults with metabolic syndrome: A 6‐month RCT. Scandinavian Med Sci Sports 2023:sms.14371. https://doi.org/10.1111/sms.14371.

[2] Norha J, Sjöros T, Garthwaite T, Laine S, Saarenhovi M, Kallio P, et al. Effects of reduced sedentary time on resting, exercise and post-exercise blood pressure in inactive adults with metabolic syndrome – a six-month exploratory RCT. J Hum Hypertens 2024. https://doi.org/10.1038/s41371-024-00894-6.

[3] Sjöros T, Vähä-Ypyä H, Laine S, Garthwaite T, Lahesmaa M, Laurila SM, et al. Both sedentary time and physical activity are associated with cardiometabolic health in overweight adults in a 1 month accelerometer measurement. Sci Rep 2020;10:20578. https://doi.org/10.1038/s41598-020-77637-3.

[4] Sjöros T, Laine S, Garthwaite T, Vähä-Ypyä H, Löyttyniemi E, Koivumäki M, et al. Reducing Sedentary Time and Whole-Body Insulin Sensitivity in Metabolic Syndrome: A 6-Month Randomized Controlled Trial. Medicine & Science in Sports & Exercise 2023;55:342–53. https://doi.org/10.1249/MSS.0000000000003054.

Supplementary Table 1. Cross-sectional correlations at baseline among all study participants.

|  |  | Physical functioning | Role functioning: physical | Role functioning: emotional | Vitality | Emotional well-being | Social functioning | General health | Pain | PSQ index | Workability score | GHQ score |
| --- | --- | --- | --- | --- | --- | --- | --- | --- | --- | --- | --- | --- |
| SB | ρ | 0.00 | 0.05 | -0.17 | 0.03 | -0.15 | -0.20 | 0.03 | -0.17 | -0.04 | -0.12 | 0.01 |
|  | P-value | 0.974 | 0.712 | 0.190 | 0.816 | 0.244 | 0.114 | 0.801 | 0.167 | 0.777 | 0.331 | 0.935 |
| Standing | ρ | -0.07 | -0.06 | 0.14 | -0.06 | 0.19 | 0.13 | -0.10 | -0.03 | 0.01 | 0.07 | -0.02 |
|  | P-value | 0.606 | 0.620 | 0.276 | 0.633 | 0.136 | 0.300 | 0.419 | 0.804 | 0.930 | 0.598 | 0.901 |
| LPA | ρ | -0.11 | 0.04 | -0.02 | 0.01 | 0.07 | 0.04 | 0.03 | 0.23 | 0.07 | 0.15 | 0.00 |
|  | P-value | 0.408 | 0.773 | 0.903 | 0.934 | 0.577 | 0.774 | 0.794 | 0.071 | 0.589 | 0.247 | 0.993 |
| MVPA | ρ | 0.15 | 0.07 | 0.17 | 0.04 | 0.04 | 0.17 | 0.15 | 0.24 | 0.00 | 0.21 | 0.05 |
|  | P-value | 0.229 | 0.565 | 0.189 | 0.772 | 0.733 | 0.192 | 0.229 | 0.059 | 0.974 | 0.099 | 0.719 |
| Steps | ρ | 0.16 | 0.04 | 0.13 | -0.04 | -0.02 | 0.14 | 0.12 | 0.24 | 0.05 | 0.17 | 0.05 |
|  | P-value | 0.208 | 0.739 | 0.312 | 0.741 | 0.863 | 0.281 | 0.348 | 0.053 | 0.705 | 0.176 | 0.688 |
| Breaks | ρ | -0.05 | 0.06 | -0.05 | -0.14 | -0.03 | -0.06 | -0.05 | 0.20 | 0.18 | 0.18 | 0.15 |
|  | P-value | 0.700 | 0.634 | 0.707 | 0.262 | 0.784 | 0.634 | 0.710 | 0.121 | 0.188 | 0.169 | 0.232 |
| VO_2_max(ml/min/kg_FFM_) | ρ | **0.32^*^** | 0.26 | -0.01 | -0.25 | -0.18 | -0.09 | 0.06 | 0.04 | **0.42^*^** | **0.31^*^** | 0.17 |
|  | P-value | **0.017** | 0.051 | 0.926 | 0.059 | 0.168 | 0.512 | 0.639 | 0.781 | **0.003** | **0.020** | 0.192 |
| VO_2_max(ml/min/kg_BM_) | ρ | **0.49^*^** | 0.24 | 0.03 | -0.08 | -0.06 | -0.01 | 0.21 | 0.14 | **0.29^*^** | 0.24 | 0.13 |
|  | P-value | **0.000** | 0.071 | 0.805 | 0.555 | 0.628 | 0.925 | 0.114 | 0.299 | **0.039** | 0.069 | 0.337 |
| BMI | ρ | **-0.25*** | -0.01 | -0.08 | 0.12 | -0.06 | 0.01 | -0.12 | -0.19 | -0.09 | 0.01 | -0.08 |
|  | P-value | **0.045** | 0.937 | 0.538 | 0.331 | 0.622 | 0.914 | 0.349 | 0.139 | 0.525 | 0.960 | 0.514 |
| Body fat % | ρ | **-0.41^*^** | -0.03 | -0.06 | -0.22 | -0.14 | -0.16 | -0.20 | **-0.30^*^** | 0.07 | -0.08 | 0.06 |
|  | P-value | **0.001** | 0.790 | 0.658 | 0.076 | 0.261 | 0.197 | 0.111 | **0.016** | 0.622 | 0.552 | 0.661 |
| Systolic BP | ρ | -0.23 | 0.03 | -0.12 | 0.06 | 0.04 | 0.10 | -0.01 | 0.12 | -0.01 | -0.03 | 0.15 |
|  | P-value | 0.068 | 0.837 | 0.363 | 0.664 | 0.768 | 0.445 | 0.938 | 0.346 | 0.915 | 0.843 | 0.248 |
| Diastolic BP | ρ | **-0.33^*^** | -0.18 | **-0.32^*^** | -0.06 | -0.05 | -0.14 | **-0.31^*^** | -0.22 | 0.26 | **-0.26^*^** | **0.27^*^** |
|  | P-value | **0.008** | 0.147 | **0.010** | 0.630 | 0.675 | 0.280 | **0.011** | 0.082 | 0.054 | **0.039** | **0.032** |
| fP-glucose | ρ | 0.02 | 0.15 | 0.06 | 0.23 | -0.02 | 0.10 | 0.01 | 0.21 | -0.20 | -0.01 | -0.16 |
|  | P-value | 0.859 | 0.232 | 0.611 | 0.063 | 0.906 | 0.446 | 0.968 | 0.095 | 0.138 | 0.960 | 0.206 |
| HbA1c | ρ | -0.15 | 0.02 | 0.07 | 0.11 | 0.01 | -0.01 | -0.04 | -0.13 | -0.06 | **-0.25^*^** | -0.05 |
|  | P-value | 0.225 | 0.872 | 0.590 | 0.396 | 0.948 | 0.909 | 0.773 | 0.313 | 0.679 | **0.044** | 0.706 |
| M-value | ρ | 0.13 | -0.01 | 0.06 | -0.17 | -0.09 | 0.05 | 0.05 | 0.18 | 0.19 | 0.16 | 0.07 |
|  | P-value | 0.328 | 0.921 | 0.612 | 0.178 | 0.486 | 0.672 | 0.671 | 0.159 | 0.165 | 0.222 | 0.557 |
| Total cholesterol | ρ | -0.20 | 0.05 | -0.15 | -0.14 | 0.02 | 0.01 | -0.03 | -0.05 | 0.17 | 0.02 | -0.05 |
|  | P-value | 0.110 | 0.692 | 0.241 | 0.261 | 0.888 | 0.921 | 0.824 | 0.679 | 0.200 | 0.848 | 0.718 |
| LDL | ρ | -0.19 | 0.03 | -0.09 | -0.09 | 0.03 | -0.08 | -0.09 | -0.12 | 0.14 | 0.09 | -0.11 |
|  | P-value | 0.146 | 0.790 | 0.483 | 0.468 | 0.825 | 0.529 | 0.503 | 0.347 | 0.290 | 0.468 | 0.408 |
| HDL | ρ | -0.18 | -0.09 | -0.11 | -0.13 | -0.06 | -0.09 | 0.03 | 0.03 | 0.05 | -0.15 | 0.10 |
|  | P-value | 0.164 | 0.489 | 0.377 | 0.309 | 0.610 | 0.476 | 0.799 | 0.793 | 0.716 | 0.236 | 0.440 |
| HDL proportion | ρ | -0.04 | -0.16 | -0.01 | -0.06 | -0.09 | -0.02 | 0.02 | 0.09 | -0.08 | -0.20 | 0.16 |
|  | P-value | 0.756 | 0.211 | 0.937 | 0.655 | 0.484 | 0.890 | 0.885 | 0.486 | 0.578 | 0.125 | 0.214 |
| fP-triglycerides | ρ | 0.03 | **0.28^*^** | 0.07 | 0.05 | 0.07 | 0.16 | 0.15 | 0.05 | -0.09 | 0.17 | -0.11 |
|  | P-value | 0.824 | **0.025** | 0.574 | 0.713 | 0.568 | 0.208 | 0.253 | 0.679 | 0.530 | 0.183 | 0.403 |

PSQ = Perceived Stress Questionnaire, GHQ = General Health Questionnaire, SB = sedentary behaviour, LPA = light physical activity, MVPA = moderate-to-vigorous physical activity, VO_2_max ml/min/kg_FFM_ = maximal oxygen uptake per kg of fat free mass, VO_2_max ml/min/kg_BM_ = maximal oxygen uptake per kg of whole body mass, BMI = body mass index, BP = blood pressure, fP = fasting plasma, HbA1c = glycated haemoglobin A1c, M-value = whole body insulin-sensitivity, LDL = low density lipoprotein cholesterol, HDL = high density lipoprotein cholesterol, HDL proportion = proportion of HDL out of total cholesterol. SB, LPA and MVPA were analysed as proportion of accelerometer wear time. *Marks statistically significant (p<0.05) correlations.

Supplementary table 2. Model-based means (95% confidence intervals) of the changes in questionnaire scores at three and six months in the intervention and control groups. The values presented correspond to figures 2 and 3.

|  | Intervention |  | Control |  |  |  |
| --- | --- | --- | --- | --- | --- | --- |
|  | Mean change at 3 mo from baseline (95% CI) | Mean change at 6 mo from baseline (95% CI) | Mean change at 3 mo from baseline (95% CI) | Mean change at 6 mo from baseline (95% CI) | P for overall group difference | P for time and sex |
| Physical functioning | -0.3 (-4.3, 3.6) | 1.1 (-3.1, 5.2) | -0.3 (-4.5, 3.9) | -2.5 (-6.7, 1.8) | 0.490 | \| Time p=0.803 \| \| --- \| \| Sex p=0.769 \| |
| Role functioning: physical | -3.3 (-13.6, 6.9) | -1.5 (-12.3, 9.4) | 1.8 (-8.9, 12.4) | -9.7 (-20.6, 1.3) | 0.811 | \| Time p=0.243 \| \| --- \| \| Sex p=0.395 \| |
| Role functioning: emotional | 2.5 (-6.9, 11.9) | -2.2 (-12.3, 7.9) | 3.3 (-6.5, 13.1) | -1.1 (-11.2, 9) | 0.871 | \| Time p=0.283 \| \| --- \| \| Sex p=0.946 \| |
| Vitality | **5.7 (1.1, 10.4)** | **5.3 (0.4, 10.3)** | **-2.9 (-7.7, 1.9)** | **-1.0 (-5.9, 4)** | **0.012*** | \| Time p=0.697 \| \| --- \| \| Sex p=0.135 \| |
| Emotional well-being | -0.8 (-5, 3.5) | 2.2 (-2.4, 6.7) | -3.9 (-8.3, 0.5) | -2.9 (-7.4, 1.7) | 0.123 | \| Time p=0.29 \| \| --- \| \| Sex p=0.061 \| |
| Social functioning | 0.6 (-3.8, 5.1) | 0.6 (-4.2, 5.4) | -1.5 (-6.1, 3.2) | -5.1 (-9.9, -0.3) | 0.154 | \| Time p=0.366 \| \| --- \| \| Sex p=0.549 \| |
| Bodily pain | 0.6 (-4.4, 5.6) | 3.7 (-1.6, 8.9) | 0.9 (-4.3, 6.1) | -4.7 (-10, 0.6) | 0.213 | \| Time p=0.487 \| \| --- \| \| Sex p=0.183 \| |
| General health | 2.4 (-1.7, 6.5) | 3.6 (-0.7, 7.9) | 0.3 (-3.9, 4.6) | -2.3 (-6.7, 2.1) | 0.123 | \| Time p=0.658 \| \| --- \| \| Sex p=0.426 \| |
| PSQ index | -0.02 (-0.061, 0.021) | -0.002 (-0.045, 0.042) | 0.015 (-0.036, 0.066) | 0.054 (0.004, 0.105) | 0.143 | \| **Time p=0.033** \| \| --- \| \| Sex p=0.810 \| |
| Workability score | -0.1 (-0.5, 0.2) | -0.1 (-0.5, 0.2) | -0.3 (-0.7, 0.1) | -0.3 (-0.7, 0.1) | 0.449 | \| Time p=0.949 \| \| --- \| \| Sex p=0.519 \| |
| GHQ score | 0.5 (-0.4, 1.3) | 0.1 (-0.8, 1.0) | 1.2 (0.3, 2.1) | 1 (0.1, 1.9) | 0.155 | \| Time p=0.380 \| \| --- \| \| Sex p=0.305 \| |

*Group difference (Tukey-Kramer) at three months p=0.012, at six months p=0.079

PSQ = Perceived stress questionnaire (higher score indicates higher stress), GHQ = General health questionnaire (higher score indicates more depressive symptoms). Adjusted for sex and baseline score.
